# Genome-Wide Association Studies in Sunflower: Towards Sclerotinia sclerotiorum and Diaporthe/Phomopsis Resistance Breeding

**DOI:** 10.3390/genes13122357

**Published:** 2022-12-14

**Authors:** Carla Valeria Filippi, Andres Corro Molas, Matias Dominguez, Denis Colombo, Nicolas Heinz, Carolina Troglia, Carla Maringolo, Facundo Quiroz, Daniel Alvarez, Veronica Lia, Norma Paniego

**Affiliations:** 1 Laboratorio de Bioquímica, Departamento de Biología Vegetal, Facultad de Agronomía, Universidad de la República, Avenida Garzón 780, Montevideo 12900, Uruguay; 2 Instituto de Agrobiotecnología y Biología Molecular–IABiMo–INTA-CONICET, Instituto de Biotecnología, Centro de Investigaciones en Ciencias Veterinarias y Agronómicas, INTA, Hurlingham B1686, Argentina; 3 Agencia De Extensión Rural General Pico, INTA, Calle 13 N° 857, Gral. Pico L6360, Argentina; 4 Estación Experimental Agropecuaria Pergamino, INTA, Av. Frondizi Km 4.5, Pergamino B2700, Argentina; 5 Estación Experimental Agropecuaria Anguil, INTA, Ruta Nacional 5 Km 580, Anguil L6326, Argentina; 6 Estación Experimental Agropecuaria Manfredi, INTA, Ruta Nac. nro. 9 km 636, Manfredi X5988, Argentina; 7 Estación Experimental Agropecuaria Balcarce, INTA, Ruta 226 Km 73.5, Balcarce B7620, Argentina; 8 Facultad de Ciencias Exactas y Naturales, Universidad de Buenos Aires, Intendente Güiraldes 2160, Ciudad Autónoma de Buenos Aires C1428, Argentina

**Keywords:** sunflower, Sclerotinia sclerotiorum, Diaporthe/Phomopsis, GWAS

## Abstract

Diseases caused by necrotrophic fungi, such as the cosmopolitan Sclerotinia sclerotiorum and the Diaporthe/Phomopsis complex, are among the most destructive diseases of sunflower worldwide. The lack of complete resistance combined with the inefficiency of chemical control makes assisted breeding the best strategy for disease control. In this work, we present an integrated genome-wide association (GWA) study investigating the response of a diverse panel of sunflower inbred lines to both pathogens. Phenotypic data for Sclerotinia head rot (SHR) consisted of five disease descriptors (disease incidence, DI; disease severity, DS; area under the disease progress curve for DI, AUDPCI, and DS, AUDPCS; and incubation period, IP). Two disease descriptors (DI and DS) were evaluated for two manifestations of Diaporthe/Phomopsis: Phomopsis stem canker (PSC) and Phomopsis head rot (PHR). In addition, a principal component (PC) analysis was used to derive transformed phenotypes as inputs to a univariate GWA (PC-GWA). Genotypic data comprised a panel of 4269 single nucleotide polymorphisms (SNP), generated via genotyping-by-sequencing. The GWA analysis revealed 24 unique marker–trait associations for SHR, 19 unique marker–trait associations for Diaporthe/Phomopsis diseases, and 7 markers associated with PC1 and PC2. No common markers were found for the response to the two pathogens. Nevertheless, epistatic interactions were identified between markers significantly associated with the response to S. sclerotiorum and Diaporthe/Phomopsis. This suggests that, while the main determinants of resistance may differ for the two pathogens, there could be an underlying common genetic basis. The exploration of regions physically close to the associated markers yielded 364 genes, of which 19 were predicted as putative disease resistance genes. This work presents the first simultaneous evaluation of two manifestations of Diaporthe/Phomopsis in sunflower, and undertakes a comprehensive GWA study by integrating PSC, PHR, and SHR data. The multiple regions identified, and their exploration to identify candidate genes, contribute not only to the understanding of the genetic basis of resistance, but also to the development of tools for assisted breeding.

## 1. Introduction

Originally from North America, sunflower (*Helianthus annuus* var. macrocarpus, Asteraceae: Heliantheae) is nowadays the fourth most important oilseed crop in the world, with approximately 57 million tons of seeds produced in 2021 [1]. It is grown throughout the world, with Ukraine, Russia, the European Union, and Argentina among the main areas of cultivation. Diseases are, and have historically been, the major limiting factors for sunflower production. There are reports of diseases caused by fungi, bacteria, viruses, mycoplasma, and parasitic plants that affect the crop [2]. However, diseases caused by fungi are by far the most widespread, and have the greatest economic impact [3]. In temperate regions of the world, diseases caused by necrotrophic fungi, such as *S. sclerotiorum* (Lib) de Bary and the Diaporthe/Phomopsis complex, are among those with the highest latent risk [4].

*S. sclerotiorum* is a cosmopolitan pathogen, capable of infecting a wide range of species (~400, from crops to weeds, [5,6]). It can attack sunflower in several ways, depending on the site and mechanism of infection. Root infection from growing fungal mycelia generates basal stem rot, while germination of aerial ascospores can generate middle stem rot and head rot (SHR, [7]). The high persistence of the inoculum in the soil in the form of long-lived sclerotia, combined with inefficient chemical control strategies and the lack of fully resistant genotypes, have made this pathogen a real threat to sunflower cultivation [8]. In fact, since the first report of *S. sclerotiorum* infestation in sunflower, the search for resistance has become one of the main breeding objectives [9]. In recent decades, the efforts of the sunflower community, especially those of public institutions, such as the Instituto Nacional de Tecnología Agropecuaria (INTA, Argentina), the Institut National de la Recherche Agronomique (INRA, France), and the United States Department of Agriculture (USDA), have succeeded in mitigating the devastating impact of this pathogen on the crop ([7,10,11,12,13,14], among others). However, losses associated with this pathogen continue to be reported.

The Diaporthe/Phomopsis complex comprises multiple species involved in disease development in sunflower, with Diaporthe helianthi being the most widespread and the first to be reported [15,16]. Of the multiple disease manifestations caused by this complex, Phomopsis stem canker (PSC) has been the most studied and most widely observed. PSC is believed to have only one primary disease cycle involving ascospores. These spores infect the margins of older leaves of sunflower plants via guttate droplets [17]. The pathogen grows up the leaf toward larger veins, continuing up the petiole to the node. The mycelium attacks parenchyma, xylem, and phloem tissue behind the node, where a characteristic stem lesion forms [15,18]. In contrast to *S. sclerotiorum*, diseases caused by Diaporthe/Phomopsis have historically had a great, but localized, impact. This way, since its initial report, it has been a major problem in Europe, where the first efforts were made to characterize the pathogen and identify sources of resistance [19,20,21]. It was also the most damaging pathogen for the crop in Uruguay [22]. In fact, after successive PSC outbreaks, sunflower ceased to be produced in this country. On the other hand, it was not a problem in the other sunflower growing regions of the world until the last decade. In 2011, Thompson et al. [23] reported on the re-emergence of the disease in Australia, showing that several species of Diaporthe/Phomopsis were implicated in the disease. A few years later, Mathew et al. reported the re-emergence of the disease in the United States [24] and Canada [25]. In Argentina, the pathogen had virtually disappeared after an initial report in 1994, which resulted in little or no losses (confirmed by frequent surveillance, [22]). However, in 2015 it re-emerged in the southern Pampas [26]. A recent study found the presence of six Diaporthe species associated with sunflower diseases in the southeast Pampas region, including D. helianthi, Diaporthe gulyae, Diaporthe kongii, Diaporthe sojae, Diaporthe caulivora, and Diaporthe longicolla [27].

Despite their different prevalence and distribution history, the two pathogens have common characteristics that make them comparable to some extent. Both pathogens generate a primary infection in the plant, caused by ascospores, existing as a strong environmental component in epidemic development. In addition, both pathogens are able to infect different parts of the plant (i.e., leaf, stem, flower head) and cause different diseases [28,29]. In the case of *S. sclerotiorum*, head rot (SHR) has the greatest damage potential, determined not only as yield reduction, but also as a decrease in the quality of the oil. As mentioned before, PSC is the most frequent disease of the Diaporthe/Phomopsis complex, although head rot (PHR) is becoming more common, at least in Argentina [30]. In both cases, variability in response (i.e., from susceptible to moderately resistant genotypes) has been reported in sunflower, with the genetic basis of resistance being complex [20]. Biparental and, more recently, association mapping studies have identified several QTL and/or genes with low to moderate effects on the phenotype for both *S. sclerotiorum* and Diaporthe/Phomopsis-associated diseases [20,28]. On the other hand, several studies reported a correlation in the response of sunflower genotypes to both diseases [20,28,31]. In this context, the question undoubtedly arises: could the resistance to these pathogens have a common genetic basis? Combined studies on the response to both pathogens have found a single common QTL [28], with very little effect, leaving the interrogant still open for identification.

In this work, we conduct a genome-wide association mapping (GWA) study for SHR, PSC, and PHR based on the association mapping population (AMP) of the INTA, Argentina [32]. In addition to conducting associations for each phenotypic trait, PCA was used to derive transformed phenotypes as inputs to a PC-GWAS. By combining signals from many traits, PC-GWAS captures the genetic signal associated with both single-trait and pleiotropic effects, resulting in increased statistical power [33]. The main objectives are (a) to identify resistance-associated markers, candidate genes, and inbred lines resistant to both pathogens that can be used in breeding programs, and (b) to contribute to the understanding of the mechanism of resistance to necrotrophic fungi in sunflower.

## 2. Materials and Methods

### 2.1. Plant Material and Phenotyping

Our AMP includes 135 inbred lines of sunflower developed and preserved by the Active Germplasm Bank of the Instituto Nacional de Tecnología Agropecuaria, Argentina (INTA). These inbred lines were selected to achieve a balance between genetic diversity [10,32,34] and adaptation to local growing conditions. In addition, these inbred lines showed a high phenotypic variability in terms of diseases (e.g., SHR, [35], Verticillium wilt, [36]), senescence [37], drought ([38] and Heinz N., personal communication), among other traits of agronomic importance. For further details on the development and initial characterization of the AMP, see Filippi et al. [32].

Information on the SHR response data used here can be found in Filippi et al. [35]. Briefly, field trials (FTs) in a randomized complete block design with two blocks and assisted inoculation were conducted in 2011, 2013, and 2014 growing seasons at the Balcarce Experimental Station INTA (37°50′0″ S, 58°15′33″ W, Buenos Aires province, Argentina). Plants were inoculated with the pathogen at the R5.2 flowering stage [39], following the method of Tourvieille de Labrouhe and Vear [9]. Disease development evaluations were performed at 14, 17, 21, 24, and 28 days post inoculation.

The response of the AMP inbred lines against SHR was estimated as disease incidence (DI), disease severity (DS), area under the disease progress curve for disease incidence (AUDPCI) and disease severity (AUDPCS), and incubation period (IP). It should be noted here that Filippi et al. [35] also conducted two other FTs (2010 and 2012 growing seasons), but these FTs were not included here because (a) in FT 2010, only 50% of inbred lines were assessed, and (b) in FT 2012, the disease levels achieved were very low. Therefore, the statistical analyses were repeated here to obtain the specific adjusted mean values for this subgroup of FTs following Filippi et al. [35].

To estimate the response of the AMP inbred lines to Diaporthe/Phomopsis infection, two successive FTs were conducted in naturally infested fields containing a range of Diaporthe/Phomopsis populations. In addition, inoculum titer was increased by aggregates of infected crop debris in the V2 stage. These FTs, consisting of a randomized complete block design with two blocks and no irrigation, were conducted in General Pico (S 35°34′43.4″ W 63°41′19.75″, La Pampa province, Argentina) in 2017 (sowing date 30 October 2017) and 2018 (sowing date 21 October 18) growing seasons. This location was selected based on reports of high incidence of the disease. The experimental unit was a row of 0.52 m in length and 5 m wide, with a planting distance of 0.52 m. Two manifestations of the disease were evaluated: Phomopsis head rot (PHR) and Phomopsis stem canker (PSC). In PHR, the symptoms are brown, rotten areas affecting the receptacles and achenes (discolored seeds), necrotic bracts, and expanded V-shaped necrosis pointing toward the peduncles. In PSC, the symptoms on the stems are pale brown cankers that develop around petiole insertions. For each of these diseases, both DI (i.e., the number of plants with symptoms on the head/stem per row) and DS (i.e., the percentage of each head/stem with symptoms) were visually registered, at the R7-R8 sunflower stage [39], on at least 10 plants per replicate. DS was scored using a scale for the stem (0, asymptomatic; 1, length smaller than 10 cm; 2, length greater than 10 cm; 3, girdling on the stem; 4, wilted or broken stems) and another for the head (0, asymptomatic; 1, lesion smaller than 10% of head area; 2, lesion between 10 and 25% of head area; 3, lesion greater than 25% of head area; 4, totally affected head), according to [40]. Graphic representations of these scales are depicted in Figure S1. For the estimation of standardized adjusted means, the proposals of Filippi et al. [35] were followed. In each case, the inbred line effect was considered as fixed, while the FT and block effects (nested in FT) were considered as random. Posteriorly, the inbred line effect was included as random in refitted models, in order to estimate the contribution of the genotype to the variance observed in the phenotypes (broad-sense heritability). Statistical analyses were conducted using InfoStat 2017 [41].

Spearman rank correlation analyses of the standardized adjusted means of all phenotypic variables, as well as the correlation plot, were performed using corrplot v0.92 [42], while principal component analysis (PCA) was performed using the prcomp function of R [43]. In addition, the k-means strategy, as implemented in NbClust v3.0.1 [44], was used to cluster the inbred lines based on their overall disease response.

### 2.2. Genotyping and GWAS

The ddRADseq sequencing data generated by Filippi et al. [34] for the AMP were retrieved and re-analyzed here to assign SNP genomic coordinates to the latest version of the sunflower genome (Han_XRQ V2.0, available at Heliagene.org). Raw sequencing data were mapped to the reference using Bowtie2 [45]. Samtools v0.1.19 [46] was used to convert to SAM/BAM format and sort the data. Then, Stacks V2.0 [47] was used in the ref_map module for SNP variant calling. Finally, VCFtools v0.1.16 [46] filtering options were applied to obtain robust SNPs. Filtering parameters included position quality > 30, allele depth > 3 reads, minor allele frequency > 0.05, and 50% as maximum percentage of missing data. In addition, a random selection was made of SNPs closer than 500 bp, with only one of them retained. Missing data were imputed using LinkImputeR v1.2.4 [48]. The generated SNP matrix can be accessed at http://github.com/cfilippi/GWAS_Han.

The association between SNPs and phenotypes was evaluated using statgenGWAS v1.0.9 [49] in R [43], following the strategy described in Kang et al. [50]. In addition to conducting a univariate GWAS for each phenotypic trait, PCA was used to derive transformed phenotypes as inputs to a univariate GWAS (PC-GWAS). To control for population structure effect, the first three PCs of a genotypic PCA were used as covariates, while an identity-by-state (IBS) kinship matrix was used to control for relatedness. Variance components were estimated using EMMA (efficient mixed model estimation), while p-values and effect size were estimated using general least squares. Following the suggestions of Ojwang et al. [51], a fixed threshold of 3 was used to determine significantly associated SNPs. In addition, the 1/n threshold (n = total number of SNPs tested for association) was applied to determine the most significant SNPs [52].

To investigate the existence of epistatic interactions between significant and non-significant SNPs, we relied on the WISH-R package v1.0 [53], which is based on the WISH method [54]. For this, SNPs were subjected to the software default quality checks. SNPs that passed the quality control were pruned using the “LD_blocks” function, considering a maximum block size of 1000 bp and a r2 threshold of 0.9. Epistatic interactions between all SNPs pairs were estimated, for each phenotype, using the “epistatic.correlation” function. Finally, all non-significant SNPs that showed a significant interaction with at least one of the associated SNPs markers (obtained from the GWA study) were retrieved.

Manhattan plots and marker effect plots were depicted using statgenGWAS v1.0.9 [49] and ggplot2 v3.3.6 [55].

### 2.3. Candidate Gene Discovery

Genes in linkage disequilibrium with the associated SNPs (i.e., with a physical distance of less than 200 Kb, considering conservative linkage disequilibrium, LD, estimates of [34]) were retrieved from the annotation file. The resulting linked genes were re-annotated using DRAGO2 [56] to predict disease resistance genes (i.e., R genes). In addition, Pannzer2 [57] was used to re-annotate the entire sunflower proteome, while topGO v2.22.0 [58] was used to predict gene enrichment within the linked genes. Finally, a literature search was conducted to identify reported regions, markers, and/or quantitative trait loci (QTL) associated with SHR and PSC/PHR response in sunflower. A co-localization study of the associated regions identified here and those reported in the literature was conducted to identify the most robust regions associated with the disease response.

## 3. Results

### 3.1. Plant Material and Phenotyping

**Figure 1:**
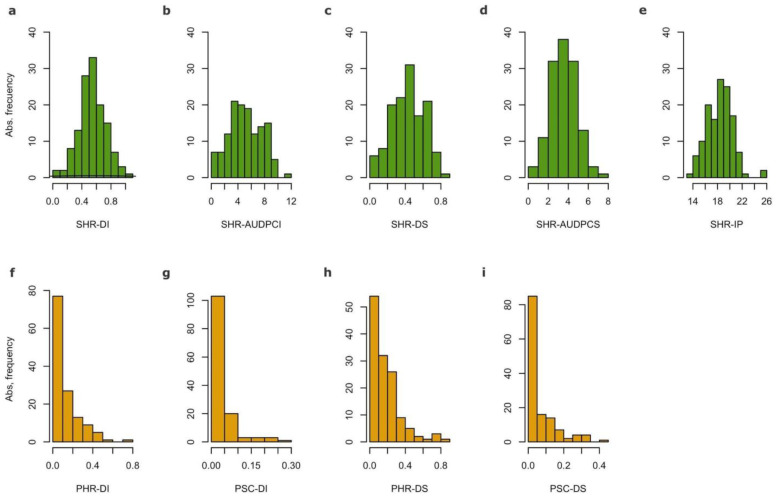
Histograms of standardized adjusted means. Upper panel: (**a**) Sclerotinia head rot (SHR) disease incidence (DI); (**b**) SHR area under the disease curve for DI (AUDPCI); (**c**) SHR disease severity (DS); (**d**) SHR AUDPC for DS (AUDPCS); (**e**) SHR incubation period (IP). Lower panel: (**f**) Phomopsis head rot (PHR) DI; (**g**) PHR DS; (**h**) Phomopsis stem canker (PSC) DI; (**i**) PSC DS.

As previously reported [35], the AMP inbred lines showed considerable variability in their response to SHR (Figure 1, upper panel, and Tables S1 and S2). On the other hand, while some variability was observed in the AMP inbred lines in their response to Diaporte/Phomopsis, only small values of PHR and PSC DI and DS were observed (Figure 1, lower panel, and Tables S1 and S2). Nevertheless, significant differences (*p* < 0.05) were observed between inbred lines. The PSC susceptible accession HA89, used as a control in other studies (e.g., [31]), showed DI and DS levels above the mean in all the FTs.

As expected, the correlation between traits recorded for the same disease was stronger than the correlation between traits for different diseases. Thus, the different traits associated with SHR showed a strong and significant correlation (positive between all traits, except IP, which showed a negative correlation with all traits), whereas DI and DS were strongly correlated in PHR and PSC. No correlation was observed for DI between PSC and PHR, but the correlation was significant for DS between PSC and PHR. This suggests that, although the incidence to both Diaporthe/Phomopsis diseases may be uncoupled, once the disease is established, the severity is subject to similar responses. Overall, the traits associated with SHR showed a weak but significant correlation with the response to PHR and PSC (*p* < 0.05). Again, this correlation was positive for all recorded traits except PI, which was negative. This can be observed in both Figure S2 and in the principal component analysis (PCA) plot in Figure S3. From the PCA analysis, it appears that responses to SHR contribute mainly to the variability captured by PC1, which explains most of the observed variance, while responses to Diaporthe/Phomopsis contribute mainly to PC2.

**Figure 2:**
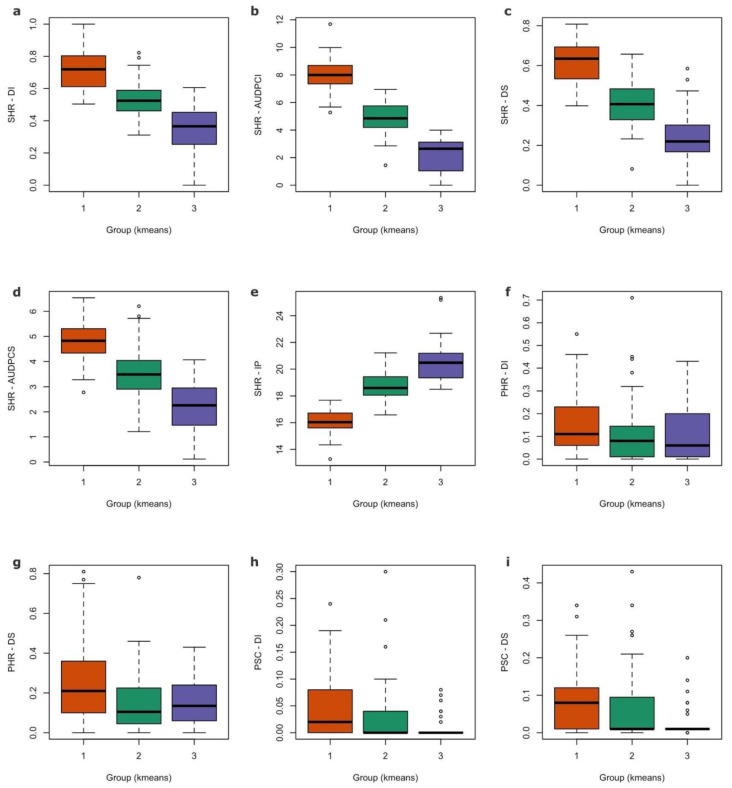
Boxplots of disease response of AMP inbred lines, discriminated by their k-means grouping (three groups): (**a**) Sclerotinia head rot (SHR) disease incidence (DI); (**b**) SHR area under the disease curve for DI (AUDPCI); (**c**) SHR disease severity (DS); (**d**) SHR AUDPC for DS (AUDPCS); (**e**) SHR incubation period (IP); (**f**) Phomopsis head rot (PHR) DI; (**g**) PHR DS; (**h**) Phomopsis stem canker (PSC) DI; (**i**) PSC DS.

The k-means analysis distinguishes three groups among the AMP inbred lines based on their general response to both diseases (Figure 2 and Figure S3). In this clustering strategy, group 3 (n = 30 inbred lines) classified the inbred lines with the best performance for both SHR (i.e., those with the lowest overall DI, AUDPCI, DS, and AUDPCS, and high IP) and PSC (i.e., those with the lowest overall DI and DS). On the other hand, the performance of the inbred lines against PHR did not differ between the defined groups (Figure 2, Table S1).

### 3.2. Genotyping and GWAS

Initially, a total of 75,433 SNPs were called against the latest version of the XRQ sunflower reference genome (V2.0, GCF_002127325.2). After initial filtering, a total of 16,705 SNPs were considered for population analysis, as PCA (to control population structure) and kinship estimation. The first PCs did not explain a high percentage of genotypic variance (PC1, 8.10%; PC2, 4.93%; and PC3, 4.48%; Figure S4). Nevertheless, and in agreement with previous reports [34,59,60], maintainer/restorer status appears to be the clearest pattern of differentiation among inbred lines.

**Figure 3:**
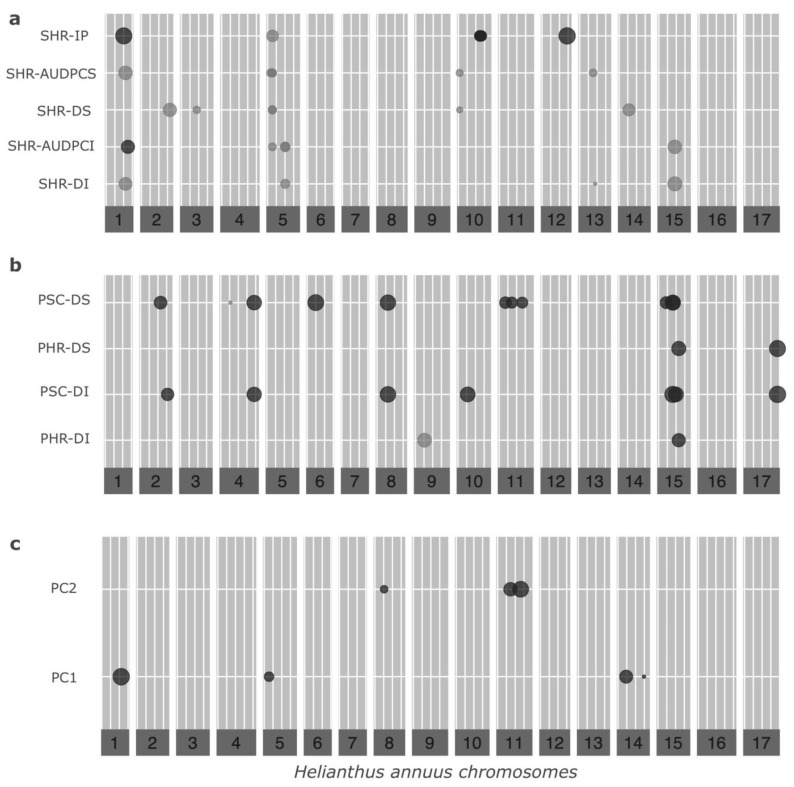
Plots of effect sizes for associated markers. Dot diameters are proportional to the effect size of the marker. (**a**) SNPs associated with phenotypic traits of SHR. DI, disease incidence; AUDPCI, area under the disease curve for DI; DS, disease severity; AUDPCS, AUDPC for DS; IP, incubation period. (**b**) SNPs associated with phenotypic traits of Diaporthe/Phomopsis. PHR, Phomopsis head rot; PSC, Phomopsis stem canker. (**c**) SNPs associated with principal components PC1 and PC2.

Subsequent elimination of strongly linked and duplicated markers resulted in 4269 SNPs that were tested for their association with phenotypes. A total of 31 marker–trait associations were found for SHR responses, of which 24 were unique to a given SHR phenotypic trait, while the remaining 7 were associated with more than 1 (Table S3, Figure 3a and Figure S5). Chromosomes CHR05 and CHR10 had the highest number of SHR-associated markers (i.e., six and eight, respectively). The effect sizes of the markers were highly variable, with the markers associated with IP in CHR01 and CHR12 showing the highest effect sizes (Figure 3). Of these associated markers, three SNPs (X791, X8416, and X13503, located on CHR01, CHR10, and CHR15, respectively) were found to be significantly associated with some phenotypic SHR traits at a more stringent threshold (1/n, Table S3).

On the other hand, 23 marker–trait associations were found for Diaporthe/Phomopsis responses, of which 19 were unique, and the remaining 4 were associated with more than one phenotypic trait of Diaporthe/Phomopsis (Table S3, Figure 3b and Figure S6). CHR15 had the highest number of these associated markers (five markers). All sunflower chromosomes, except CHR07 and CHR16, contained at least one associated marker. Four SNPs (X2192, X3761, X5326, and X9983, located on CHR02, CHR04, CHR06, and CHR11, respectively) were most significantly associated with several phenotypic PSC traits, while one (X13615, located on CHR15) was most significantly associated with both PHR DI and DS, at a more stringent threshold (1/n, Table S3).

Less variable marker effects were observed for markers associated with phenotypic traits of Diaporthe/Phomopsis when compared to markers associated with phenotypic SHR traits (Figure 3). None of the markers were simultaneously associated with phenotypic SHR and Diaporthe/Phomopsis traits.

PC-GWAS, for the first two PCs of the PCA, yielded a set of seven SNPs: four associated with PC1 and three with PC2 (Table S3, Figure 3c and Figure S7). Of these, two SNPs associated with PC1 were also associated with SHR phenotypic traits (X791 and X4048), while two SNPs associated with PC2 were also associated with Diaporthe/Phomopsis phenotypic traits (X6852 and X10160). This is in line with expectations, given that responses to SHR contribute mainly to the variability captured by PC1, while responses to Diaporthe/Phomopsis contribute mainly to PC2.

Epistatic interaction effects among significant SNP and non-significant SNP markers yielded multiple SNP pair interactions for the different phenotypic traits evaluated herein. By filtering to obtain only those cases where interactions involved the maximum number of associated SNPs for each trait, a total of 56 k-SNP (k ≥ 2) epistatic interactions emerged as candidates for further studies (Table S4). As an example, SNP X5683 showed epistatic interactions with the two markers significantly associated with response to PSC-DS (i.e., X13615 and X16661), while the marker X12761 showed epistatic interactions with 8/10 SNPs associated with SHR-IP. While most of the non-significant SNPs fall within non-coding regions, seven fall within genes, including a receptor-like kinase (RLK-46, marker X5683).

### 3.3. Candidate Gene Discovery

**Figure 4:**
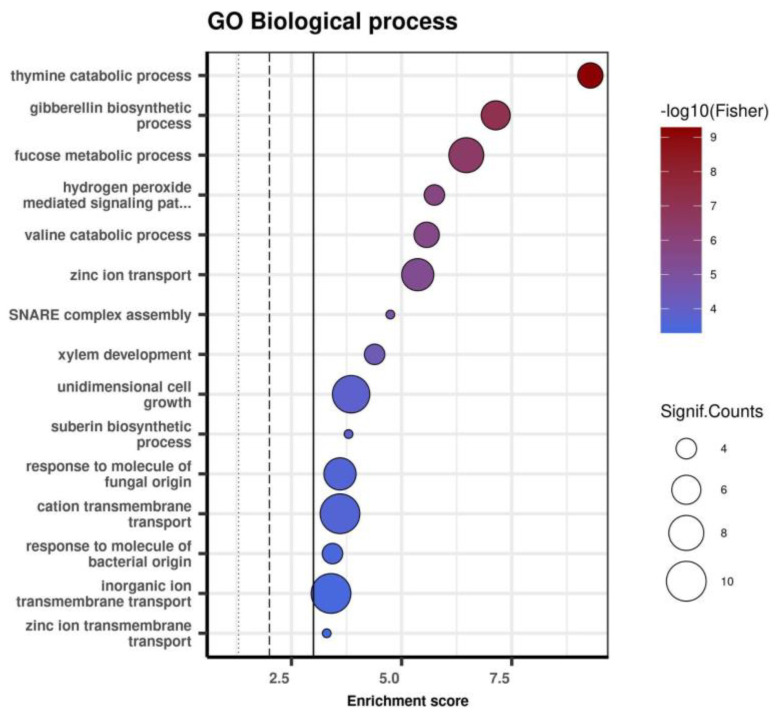
GO biological process terms enriched in the 364 genes physically close to the associated markers (only the first 15 major terms are shown).

Examination of the 200 Kb region around each significantly associated SNP allowed the identification of 364 physically close genes. Gene enrichment analysis showed that biological processes, such as hydrogen peroxide signaling pathways, response to molecules of fungal origin, response to molecules of bacterial origin, and others, were significantly enriched in the 364 genes physically closest to the associated markers (Figure 4). The GO enrichment results for the “molecular function” and “cellular component” aspects are presented in Figure S8.

In addition, prediction of disease resistance genes using DRAGO2 [56] revealed 19 putative disease resistance genes in these 364 genes. These genes were physically located near 13 of the 43 associated SNPs (9 associated with SHR response, while the remaining 4 were associated with Diaporthe/Phomopsis response, Table 1). In addition, two of the eight most significantly associated SNPs were located near a disease resistance gene. Most of the disease resistance genes identified by DRAGO2 belong to the kinase family, and only one (LOC110882123, associated with SHR response) is a nucleotide-binding site receptor.

## 4. Discussion

The devastating and cosmopolitan *S. sclerotiorum*, and the re-emerging *Diaporthe*/*Phomopsis* complex are currently the necrotrophic fungi with the greatest impact on global sunflower production. Although natural variability is observed, the lack of complete resistance combined with the inefficiency of chemical control has made assisted breeding the best disease control strategy. In this work, we present an integrated study of the response of a diverse panel of sunflower inbred lines, the AMP of the INTA (Argentina), to both fungal pathogens.

Disease levels achieved for SHR were consistent across field trials (FTs). In contrast, disease levels for *Diaporthe*/*Phomopsis* were erratic and tended to be low for both DI and DS. This fact reflects the differences in the way the FTs were conducted. SHR FTs were conducted with irrigation and assisted inoculation to support conditions for ideal infection. Indeed, traits evaluated after artificial inoculation usually show higher heritabilities than those obtained in semi-natural infection conditions [61]. In the absence of a suitable assisted inoculation method for large-scale FTs, *Diaporthe*/*Phomopsis* FTs were performed in naturally infested fields. One of the major drawbacks of relying on non-assisted infection is the high dependence not only on the availability of a viable inoculum, but also on the occurrence of climatic conditions that allow the disease to establish and develop [62].

*Diaporthe*/*Phomopsis* benefits from abundant moisture and temperatures between 23–25 °C during the growing stage [2]. In the FTs conducted here, the first one (growing season 2017) allowed higher disease levels, while in the second FT, high temperatures and scarce rains prevented full disease development (growing season 2018). The registered disease levels allowed a significant differentiation between inbred lines, and were in the same range as those reported by Talukder et al. [31] for PSC in four FTs also conducted with non-assisted infection. However, the impact of the environment on genotypes was higher in our study than in Talukder et al. [31]. This also became apparent in the lower broad-sense heritability values observed herein, when compared to those reported by Talukder et al. [31]. On the other hand, one of the main advantages of performing FTs under non-assisted infection is that it allows the evaluation of multiple manifestations of the disease (i.e., PSC and PHR). In addition, it is worth pointing out that the currently reported artificial infection methods for *Diaporthe*/*Phomopsis* involve inoculation with fungal mycelium. While these methods allow the study of the kinetics of symptom development, they cannot reveal the resistance to penetration by ascospores.

Analysis of the genotypic matrix revealed the importance of hard filtering SNP data for good data quality. Here, only ~5.7% of the original SNP matrix was considered for marker–phenotype association testing (i.e., 4269 out of 75,433 SNPs). The main reasons for filtering out the markers were the high percentage of missing data, low MAF, and the presence of strongly linked markers. This was not unexpected as the strategy used for data generation, ddRADseq, typically results in a high percentage of missing data [63]. Furthermore, this restriction-associated sequencing strategy achieves an almost binomial distribution of marker distance (i.e., near vs. far): while SNPs identified on the same read have physical distances of less than 100 bp, SNPs from different sequenced reads can be many hundreds of bp apart. In this way, filtering for “only one SNP per 300 bp window” allowed a substantial reduction in SNP number, with only informative SNPs considered for marker–phenotype association tests.

The GWAS analysis revealed 24 unique marker–trait associations for SHR and 19 unique marker–trait associations for *Diaporthe*/*Phomopsis*, with CHR05, CHR10, and CHR15 having the highest number of associated loci. No common markers were found for the response to the two pathogens studied. This is not unexpected considering the complex genetic base of the resistance, the size of our AMP (which only allows the detection of strong associations), and the weak correlation between phenotypic SHR and PSC traits. The observed correlation values are comparable to those reported by Bert et al. [20] for *S. sclerotiorum* and *Diaporthe*/*Phomopsis* phenotypic traits (*r*~0.20). In an independent work, Talukder et al. [31] reported a moderate correlation between SHR and PSC (*r* = 0.52). However, the GWA study conducted on the basis of Talukder’s phenotypic data, and using > 200,000 SNPs as genotypic data, found only a single common associated region for SHR and PSC [28]. These weak correlations, coupled with little or no overlap of resistance-associated regions, may indicate relative independence of the response to SHR and *Diaporthe*/*Phomopsis* in sunflower.

As a complementary approach, a GWAS using PC scores as dependent variables was performed. The effectiveness of PC-GWAS relies on (a) the ability of this strategy to decrease the type I error rate [64]; (b) the transformation of skewed variables to an approximately normal distribution [65]; and (c) the fact that the use of PCs allows the detection of genomic regions using individual traits to be overlooked, since PC scores represent integrated variables [66]. Using the PC scores for GWAS, we identified significant associations with PCs; including SNPs simultaneously associated with a single trait, along with others that were considered novel.

While each SNP associated via GWA is expected to have a significant effect in determining a phenotypic trait, non-significant markers interacting with these associated SNPs could also have an influence on such traits [67]. Considering epistatic interactions constitutes a complementary approach to the study of GWA, allowing the discovery of potentially associated markers, with impact on phenotype [68]. In this regard, while no common associated markers were identified via GWA between the two pathogens responses, the study of epistatic interactions allowed the identification of the marker X16659, which was significantly associated with PSC-DI and showed epistatic interactions with 7/10 markers associated with SHR-IP. X16659 is located in chromosome 17, in LD with LOC110926506, an RLK. Similarly, the marker X9256, significantly associated via GWA with SHR-IP, showed epistatic interactions with two markers associated with PSC-DS (X13615 and X16661). This suggests that, while the main determinants of resistance may differ for the two pathogens, there could be an underlying common genetic basis, thus explaining the observed correlations.

The search for candidate genes in the regions underlying the associated SNPs revealed several genes of interest based on their putative function. Prediction of resistance (R) genes using a tool developed specifically for this purpose (DRAGO2, [56]) yielded 19 putative R genes. Of these, only one belongs to the nucleotide-binding and leucine-rich repeat (NLR) family of cytoplasmic receptors. The other identified R genes belong to the so-called pattern recognition receptors (PPRs), which are proteins that recognize pathogen-associated molecules (PAMPs). The small number of NLRs observed makes sense, as these types of receptors are more commonly associated with qualitative (i.e., gene-to-gene) resistance, in which a receptor recognizes a race-specific effector triggering a hypersensitive response. Indeed, approximately 30% of identified NLRs are located on CHR13 of sunflower [69]. However, there are still no reports of QTLs with major effects or clusters of loci associated with resistance to *S. sclerotiorum* and/or *Diaporthe*/*Phomopsis* on this chromosome ([11,12,20,28,70,71], among others).

Quantitative resistance, as observed in *S. sclerotiorum* and *Diaporthe*/*Phomopsis* responses, is more commonly associated with broad-spectrum genes such as PPRs. The most common PPR class observed herein was receptor kinase or RLK. RLKs are membrane-associated receptors capable of recognizing PAMPs and triggering a defense response. However, not all members of the multi-family kinase are involved in defense processes. On the contrary, these proteins play a role in almost all biological functions [72]. Although considerable progress has been made in the study and characterization of sunflower kinases, a complete functional classification and global analysis of the expression patterns of this large gene family are still lacking. In addition to the putative candidate genes for resistance, other important genes were identified in the surrounding regions of the associated markers. Among them, we highlight a gene encoding a germin-like protein (GLP15, LOC110898542, near to SNP X11750, associated with SHR-DI). Several studies demonstrate the involvement of germin and GLPs, together with oxalate oxidase/superoxide dismutase activity, in defense mechanisms against *S. sclerotiorum* infections in several species, including sunflower (e.g., [10,73,74,75]). Calcium-dependent genes have also been identified (LOC110890454, close to SNP X10160, and LOC110926044, near to SNP X9983, both associated with PSC-DS). This is of particular interest because calcium acts as a second messenger for signaling a variety of stimuli, including PAMPs [76]. Changes in calcium concentration are sensed by proteins, such as calmodulin, which affects the plant’s ability to respond to pathogens [77]. Interestingly, LOC110926044 was reported as differentially expressed in sunflower after artificial inoculation with *S. sclerotiorum* [78], in the partially resistant inbred line RK416. In addition, a gene encoding a putative Rho-associated protein was also identified (LOC110920635, near SNP X1907, associated with PSC-DS). The association of a haplotype of a gene encoding a putative Rho-interactive protein (rhoBP_B) with a decrease in SHR-DI was previously reported by Fusari et al. [10]. Thus, while the role of this family in the defense process remains to be elucidated, there is cumulative evidence for the involvement of these proteins in the defense response.

This paper presents the first simultaneous large-scale evaluation of two manifestations of *Diaporthe*/*Phomopsis* (i.e., PSC and PHR) in sunflower. The combined evaluation of phenotypic data obtained for PSC and PHR, together with data previously generated for SHR [35], allowed the identification of a group of inbred lines with good general behavior against these pathogens that can be used as donors in breeding programs. In addition, several regions associated with these diseases have been identified, contributing not only to the understanding of the genetic basis of resistance, but also to the development of tools (i.e., QTL and molecular markers) for assisted breeding. Future challenges include the development of an appropriate assisted inoculation method for large-scale FTs, to support conditions for ideal infection, and the development of a comprehensive picture of the prevalence of the various *Diaporthe*/*Phomopsis* species causing PHR and PSC at our screening site over the years (Colombo D., personal communication). We anticipate that these studies will have a concrete application in the near future.

## 5. Conclusions

The complex nature of the genetic basis of sunflower resistance to diseases caused by *S. sclerotiorum* and the *Diaporthe*/*Phomopsis* complex is evidenced by the multiple associated SNPs found, in addition to the identification of epistatic interactions. Although no common SNPs were found to be associated with resistance to both pathogens, the study of epistatic interactions allowed the identification of two markers significantly associated with the response to *S. sclerotiorum* and *Diaporthe*/*Phomopsis*. The posterior search for genes physically linked to the associated SNPs revealed in all cases pathogenesis-related genes, mainly of the “receptor-like kinase” class. In-depth analysis of these genes may help to dissect the underlying mechanism of resistance to both pathogens in sunflower. It is expected that the associated regions, and the identified sources of resistance, will contribute to the development of tools for assisted breeding in crops.

## Figures and Tables

**Table 1:** Disease resistance candidate genes physically located near associated markers.

Associated SNP	Chromosome	SNP Position (bp)	Disease Resistance Genes ID ^1^	Class ^2^	Associated Trait
X2277	2	163473796	LOC110926750	RLP	SHR-DS
X2732	3	88494320	LOC110929054	CK	SHR-DS
X4003	5	17142118	LOC110938599, LOC118479338, LOC110938600, LOC110942752	KIN, KIN, KIN, KIN	SHR-AUDPCS
X4045	5	27222485	LOC110938841	KIN	SHR-DS, SHR-AUDPCS
X4048	5	27237353	LOC110938841,	KIN	SHR-DS, SHR-AUDPCI, SHR-AUDPCS, PC1
X4053	5	28040371	LOC110940617, LOC110940627	KIN, KIN	SHR-IP
X9267	10	123241420	LOC110882123	N	SHR-IP
**X9983**	**11**	**36101150**	**LOC110926271**	**CK**	**PSC-DS**
X11154	12	143897929	LOC110895591	KIN	SHR-IP
X13326	15	42971601	LOC110911446, LOC110911384, LOC110911385	KIN, KIN, KIN	PSC-DS
X13006	15	148107847	LOC110904282	KIN	**PC1**
**X13502**	**15**	**91884882**	**LOC110881738, LOC110881737**	**KIN, KIN**	**SHR-DI**
X16659	17	187789299	LOC110926506	KIN	PSC-DI
X16661	17	187789629	LOC110926506	KIN	PHR-DS

^1^ Gene IDs are those defined in NCBI (GCF_002127325.2). ^2^ Disease resistance classes are those defined by Osuna-Cruz et al. [56]. SHR, Sclerotinia head rot; PHR, Phomopsis head rot; PSC, Phomopsis stem canker; DI, disease incidence; AUDPCI, area under the disease curve for DI; DS, disease severity; AUDPCS, AUDPC for DS; IP, incubation period. In bold, markers significant at the 1/n threshold. Marker significance, additive effect, and others, are listed in Table S3.

## Data Availability

The generated SNP matrix can be accessed at http://github.com/cfilippi/GWAS_Han.

## Supplementary Materials

The following supporting information can be downloaded at https://www.mdpi.com/article/10.3390/genes13122357/s1. Figure S1: Scales used for disease severity assessment in (a) Phomopsis stem canker (PSC) and (b) Phomopsis head rot (PHR). Values (0–4) are those described in the methodology section. Figure S2: Spearman’s correlation analysis of Sclerotinia and Phomopsis head rot (SHR and PHR) and Phomopsis stem canker (PSC)-related variables. Figure S3: Principal component analysis biplot based on the adjusted means of Sclerotinia and Phomopsis head rot (SHR and PHR) and Phomopsis stem canker (PSC)-related variables. Points represent inbred lines. Variables are represented as lines extending from the center of the biplot. Inbred lines are colored based on their group assignment. Figure S4: Principal component analysis biplot for the 135 sunflower inbred lines, assessed with 16,705 SNPs. Inbred lines are colored according to their maintainer/restorer status (HA vs. RHA). N/A: accessions for which no information was available. Figure S5: GWAS Manhattan plots for (a) Sclerotinia head rot (SHR) disease incidence (DI); (b) SHR area under the disease curve for DI (AUDPCI); (c) SHR disease severity (DS); (d) SHR AUDPC for DS (AUDPCS); (e) SHR incubation period (IP). Figure S6: GWAS Manhattan plots for (a) Phomopsis head rot (PHR) disease incidence (DI); (b) PHR disease severity (DS); (c) Phomopsis stem (PSC) canker DI; (d) PSC DS. Figure S7: PC-GWAS Manhattan plots for (a) principal component 1 (PC1) and (b) principal component 2 (PC2). Figure S8: GO terms enriched in the 364 genes physically close to the associated markers (only the first 15 major terms are shown): (a) molecular function, (b) biological process, (c) cellular component. Table S1: Standardized adjusted means for the phenotypic traits evaluated herein. Table S2: General statistics of the field trials. Table S3: General characteristics of associated markers. Table S4: Results of the k-SNP (k ≥ 2) epistatic interactions identified herein.
