# Supplementary figures and images for: Genome-Wide Association Studies in Sunflower: Towards Sclerotinia sclerotiorum and Diaporthe/Phomopsis Resistance Breeding
Source: Genes (Basel). 2022 Dec 14;13(12):2357. doi: 10.3390/genes13122357 (PMC9777803; doi:10.3390/genes13122357)

a)

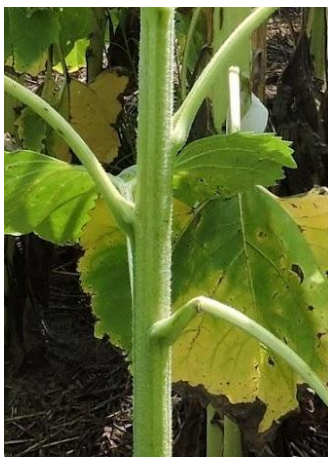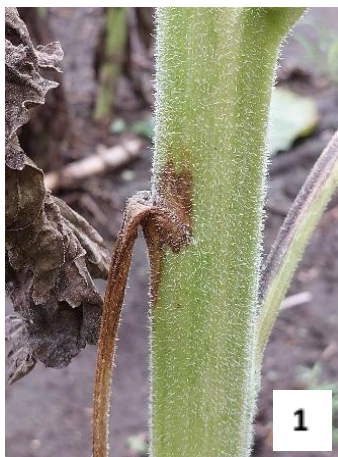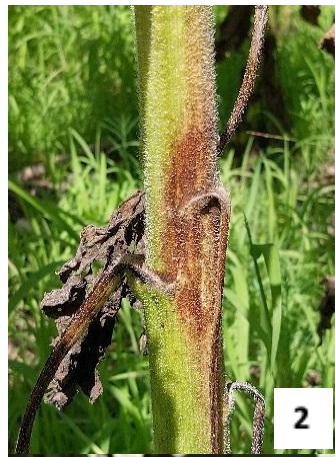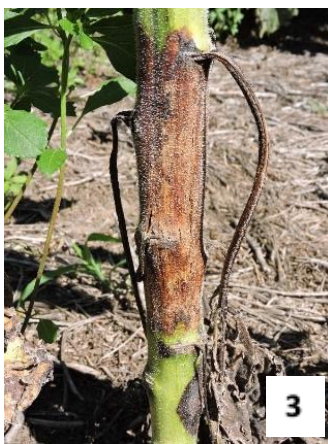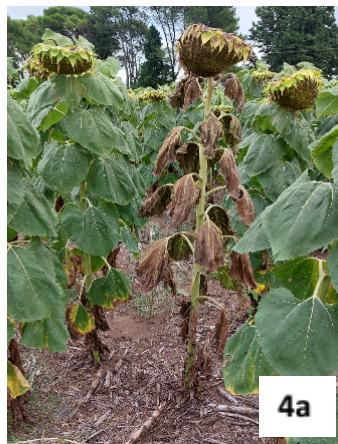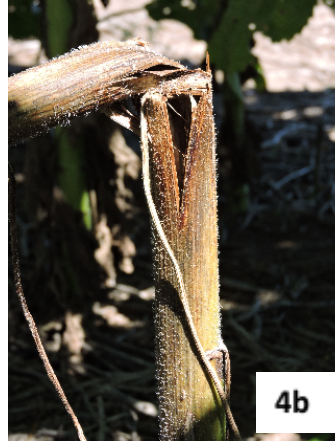

b)

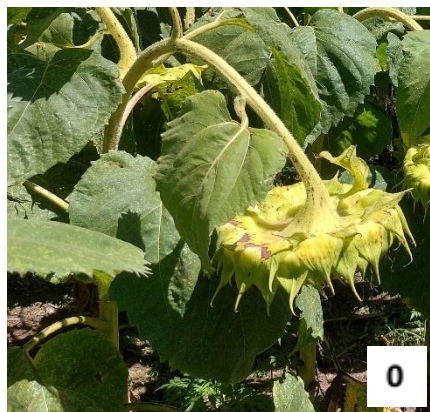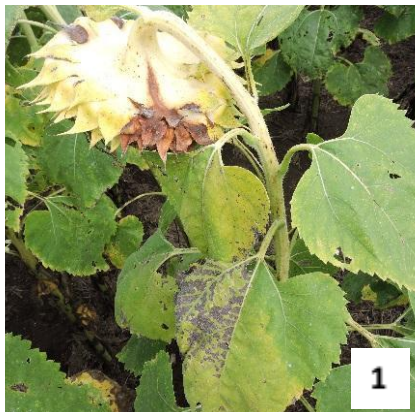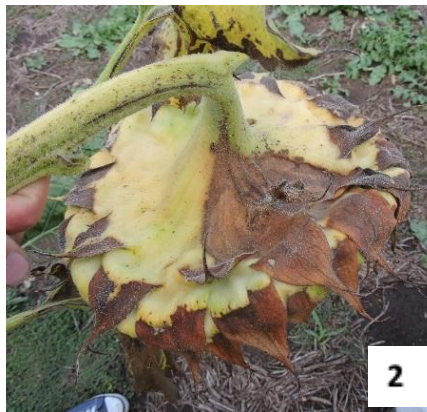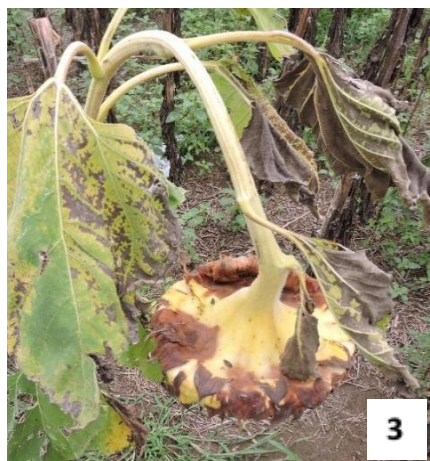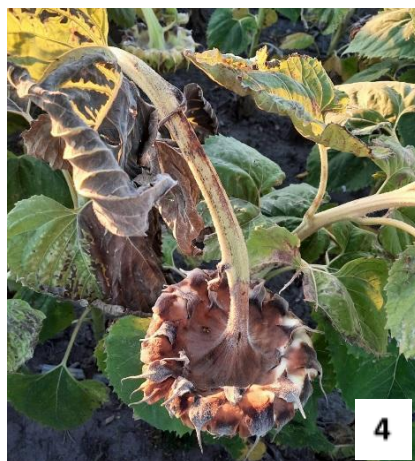

Supplement: Supplementary file 1 [file genes-13-02357-s001.zip › Figure_S1.pdf]

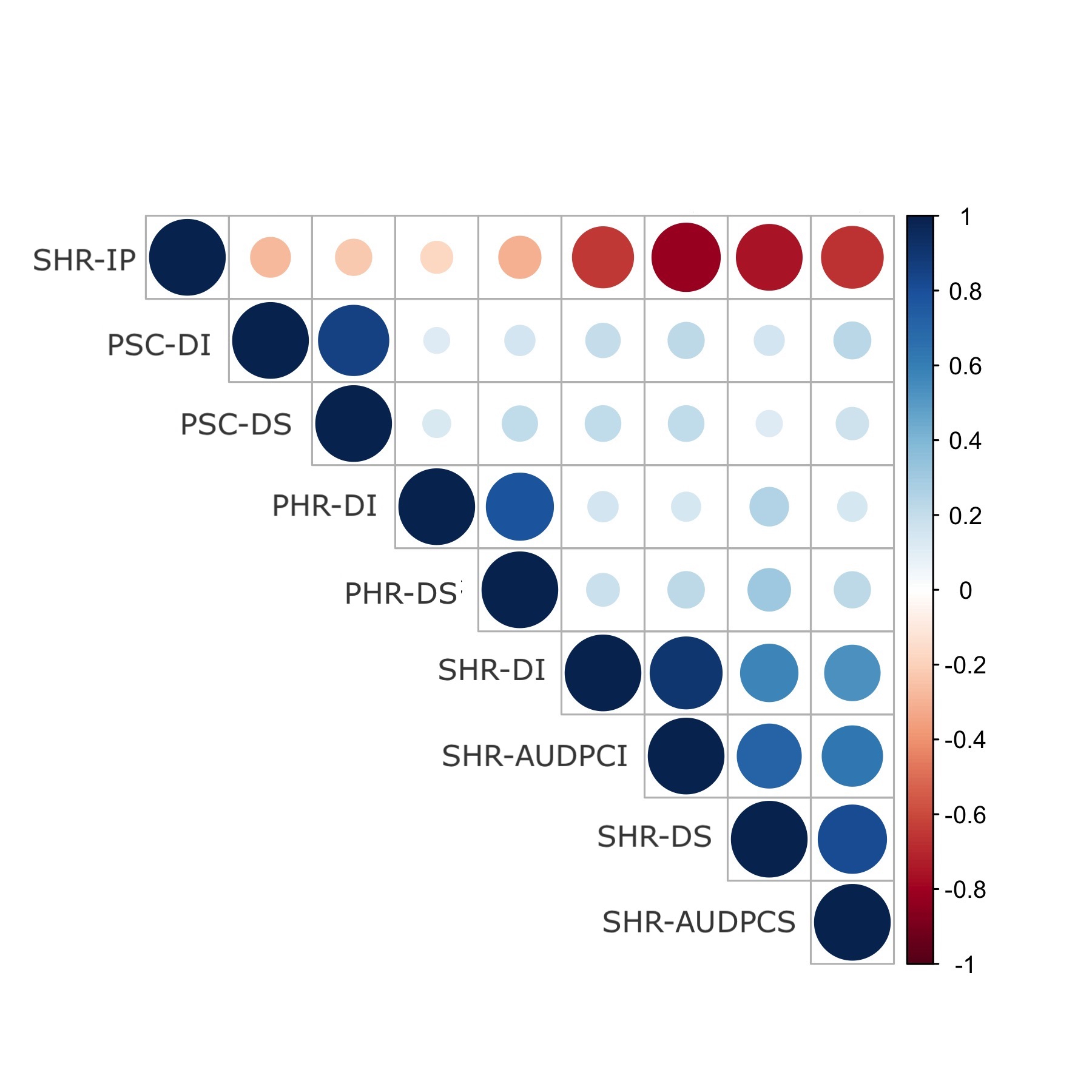

Supplement: Supplementary file 1 [file genes-13-02357-s001.zip › Figure_S2.jpg]

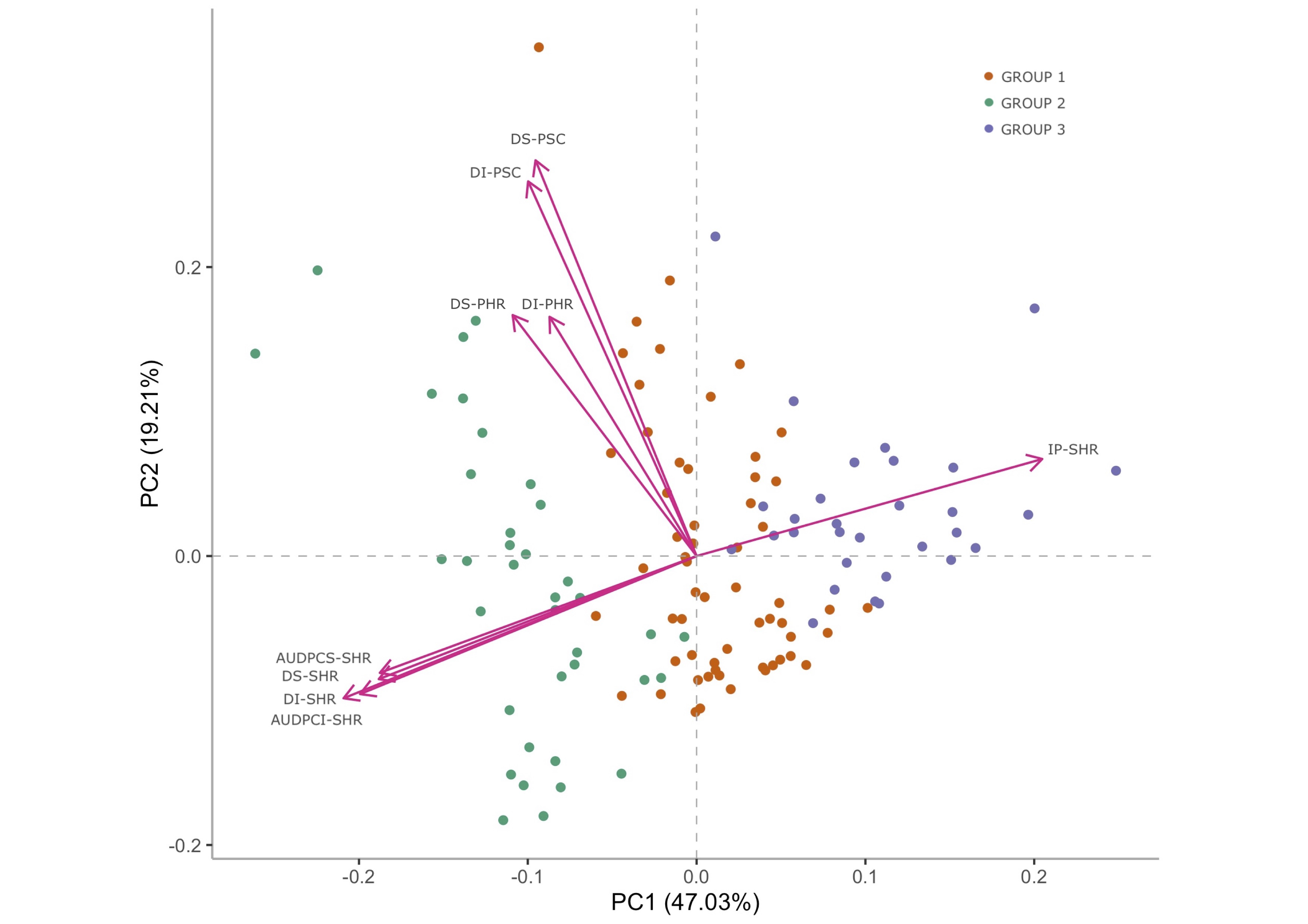

Supplement: Supplementary file 1 [file genes-13-02357-s001.zip › Figure_S3.jpg]

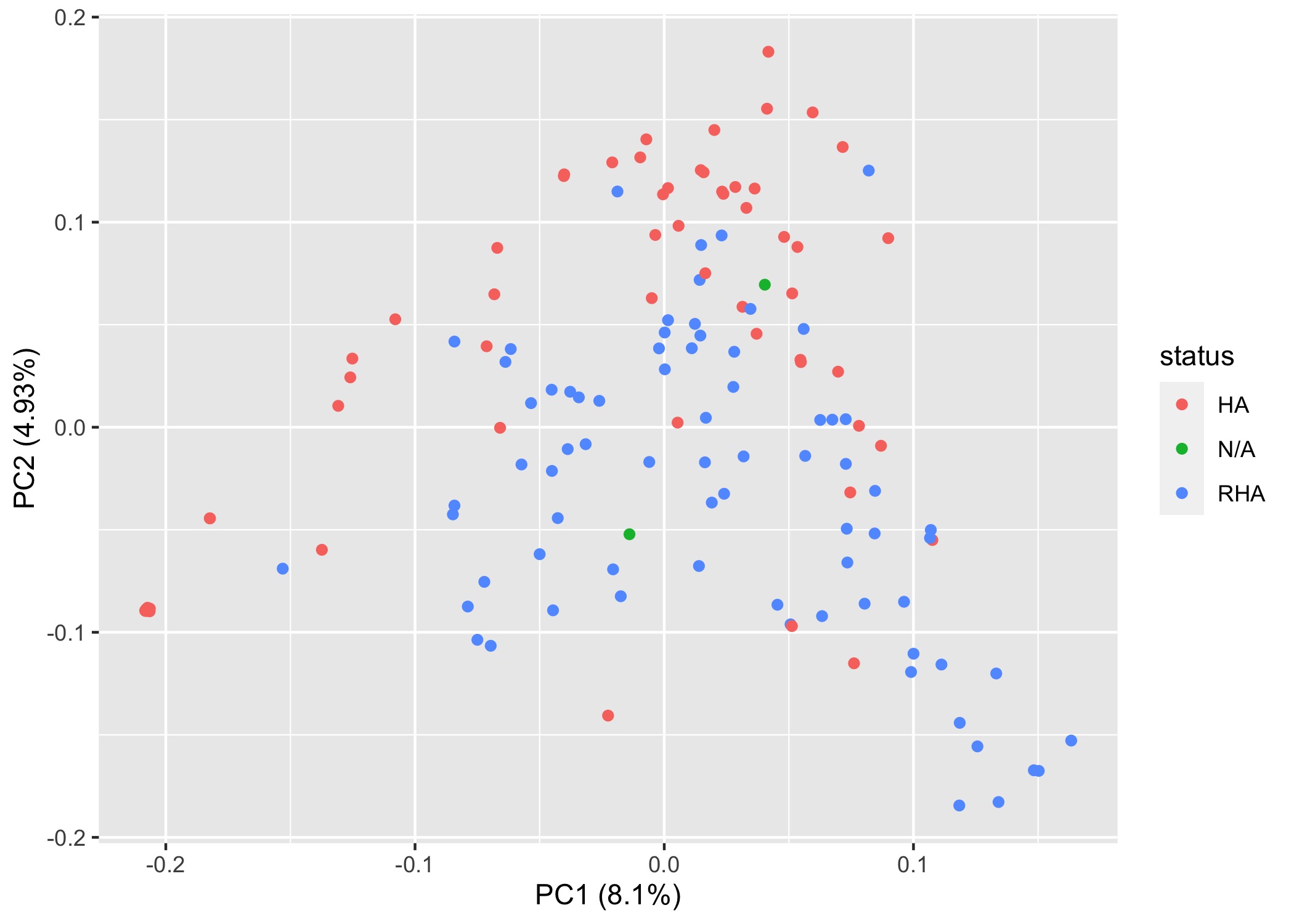

Supplement: Supplementary file 1 [file genes-13-02357-s001.zip › Figure_S4.jpeg]

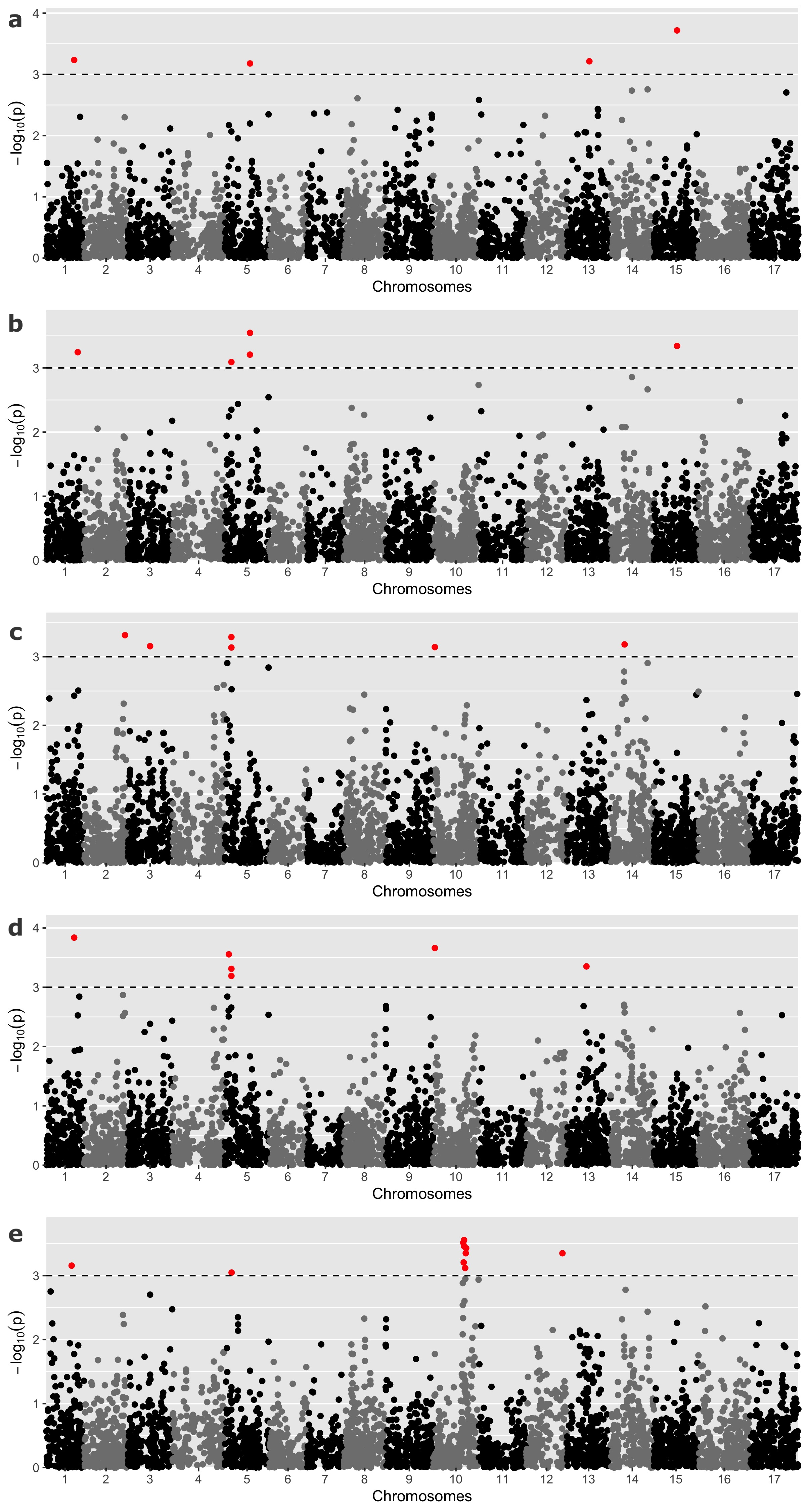

Supplement: Supplementary file 1 [file genes-13-02357-s001.zip › Figure_S5.jpg]

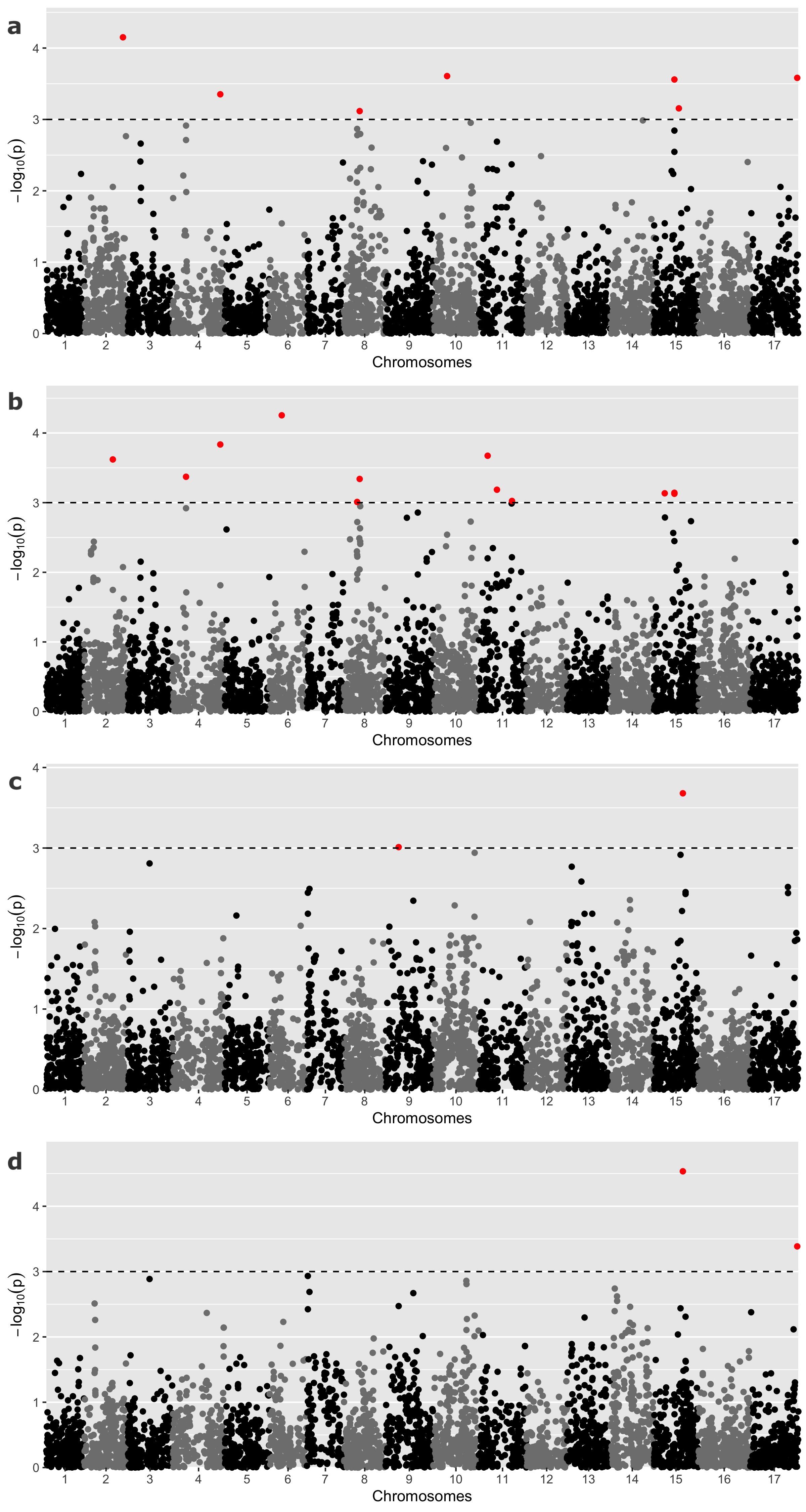

Supplement: Supplementary file 1 [file genes-13-02357-s001.zip › Figure_S6.jpg]

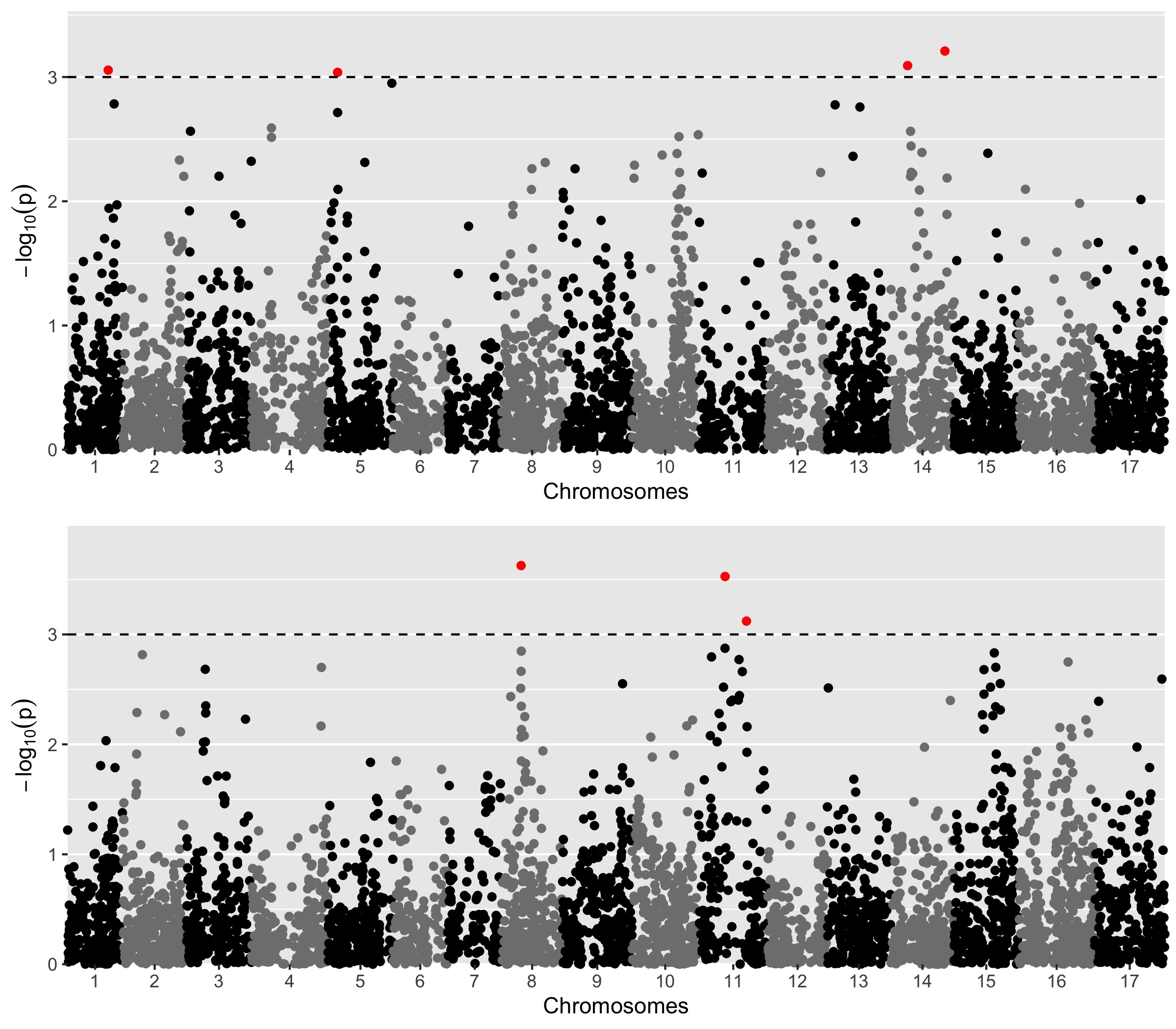

Supplement: Supplementary file 1 [file genes-13-02357-s001.zip › Figure_S7.jpeg]

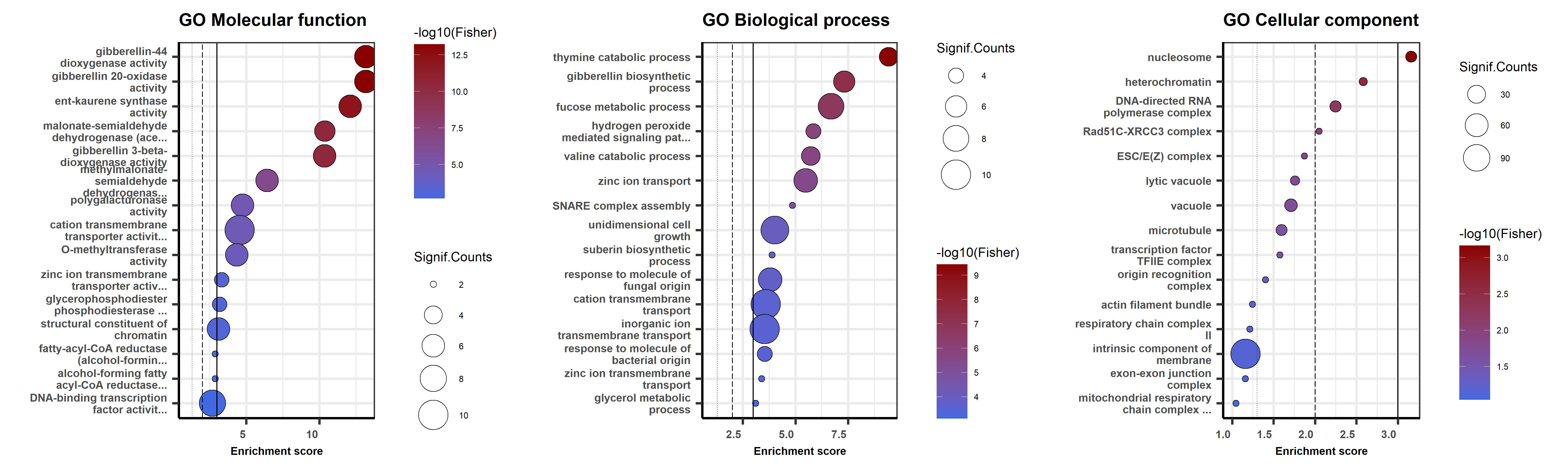

Supplement: Supplementary file 1 [file genes-13-02357-s001.zip › Figure_S8.jpg]
